# High PROX1 expression in gastric cancer predicts better survival

**DOI:** 10.1371/journal.pone.0183868

**Published:** 2017-08-30

**Authors:** Alli Laitinen, Camilla Böckelman, Jaana Hagström, Arto Kokkola, Pauliina Kallio, Caj Haglund

**Affiliations:** 1 Department of Surgery, University of Helsinki and Helsinki University Hospital, Helsinki, Finland; 2 Research Programs Unit, Translational Cancer Biology, University of Helsinki, Helsinki, Finland; 3 Department of Pathology and Oral Pathology, University of Helsinki and Helsinki University Hospital, Helsinki, Finland; University of South Alabama Mitchell Cancer Institute, UNITED STATES

## Abstract

**Background:**

PROX1 is a transcription factor involved in the development of various organs. It has also an important function in colorectal cancer progression. The aim of this study was to investigate the prognostic role of PROX1 expression in gastric cancer.

**Methods:**

We evaluated PROX1 expression in gastric cancer by immunohistochemistry of tumor-tissue microarrays including tumor specimens from 283 patients who underwent surgery at Helsinki University Hospital. We investigated the association of PROX1 expression with clinicopathologic variables and patient survival.

**Results:**

Cytoplasmic PROX1 reactivity was high in 56 (20.5%) and low in 217 (79.5%) cases. Low PROX1 immunostaining associated with diffuse cancer type (p = 0.002). In subgroup analysis, PROX1 was a significant marker of better prognosis in patients aged under 66 (p = 0.007), in those with intestinal cancer (p = 0.025), among men (p = 0.019), and in tumors of less than 5 cm diameter (p = 0.030). Patients with high PROX1 expression had a cancer-specific 5-year survival of 65.6% (95% CI 52.7–78.5), compared to 37.1% (95% CI 30.2–44.0) for those with low expression (p = 0.004, log-rank test). This result remained significant in multivariable analysis (HR = 0.56; 95% CI 0.35–0.90; p = 0.017).

**Conclusion:**

In gastric cancer, high cytoplasmic PROX1 expression is an independent marker of better prognosis.

## Introduction

Gastric cancer is a highly malignant tumor and is globally one of the major causes of cancer-related death [1]. Its incidence in the last decades has decreased, but its 5-year survival rate, despite curative surgery is, in the Western world, still less than 30% [2]. Besides the established prognostic factors such as TNM-stage together with the possibility of curative surgery, additional prognostic information may come from tumor markers.

The transcription factor PROX1 is a key regulatory protein in the development of various organs. It is involved in cell-fate determination and progenitor-cell regulation in the central nervous system, eye, liver, pancreas, lymphatic system, and the heart. It also plays a role in cancer development. Previous studies have indicated that PROX1 can have either tumor-suppressive or oncogenic properties. Various levels of PROX1 protein occur in tumor cells, but the role of PROX1 in the proliferation, migration, and invasion of cancer cells is unclear [3].

PROX1 is involved in various gastrointestinal tract tumors [4–10], in hematological malignancies [11], in breast cancer [12], in Kaposi sarcoma [13], and in brain tumors [14,15]. In colorectal cancer, PROX1 plays an essential role in tumor progression; high immunohistochemical nuclear PROX1 expression is associated with poor patient outcome [4,5]. Conversely, in hepatocellular carcinomas [6], in pancreatic ductal adenocarcinomas [8,9], and in carcinomas of the biliary system [10], low PROX1 expression is associated with poor prognosis. In esophageal squamous cell carcinoma cells, over-expression of PROX1 inhibits tumor cell proliferation [7].

In gastric cancer, PROX1 may promote tumor progression by inducing cancer-cell proliferation and lymphangiogenesis [16]. PROX1 expression when, appearing in gastric cancer tissue, may serve as a potential prognostic factor and treatment target. Moreover, tumorigenesis and tumor progression is also linked to dysregulation of microRNAs. Recently Zhang et al. [17] found that miR-489 was significantly downregulated in human gastric cancer tissues and cell lines, and PROX1 was a direct miR-489 target.

The aim of this study was to determine the prognostic value of immunohistochemical staining of PROX1 in gastric cancer.

## Materials and methods

### Patients

In total, 313 patients underwent surgery for histologically verified gastric adenocarcinoma at the Department of Surgery, Helsinki University Hospital between 2000 and 2009. We included 283 tissue samples from patients undergoing total or partial gastrectomy with D1- or D2-lymphadenectomy, 228 (72.8%) of them with curative intent, whereas 85 (27.2%) underwent palliative surgery. Median age was 67.4 (interquartile range 57.1–76.5); 161 (51.4%) were women. Stage distribution according to the 7^th^ edition of the UICC classification was 62 (19.9%) stage IA-IB, 72 (23.1%) stage IIA-IIB, 115 (36.8%) stage IIIA-IIIC, and 63 (20.2%) stage IV patients. Lymph-node metastases occurred in 198 (65.6%) and distant metastases in 63 (20.1%). Of the 313 patients, 15 (4.8%) received neoadjuvant treatment, and 125 (39.9%) postoperative adjuvant treatment (74 chemotherapy, 2 radiotherapy, and 50, both). Survival data and causes of death until October 2016 came from patient records, the Population Register Centre of Finland, and Statistics Finland.

The study was approved by the Surgical Ethics Committee of Helsinki University Hospital (Dnro HUS 226/E6/ 06, extension TMK02 §66 17.4.2013) and the National Supervisory Authority of Welfare and Health gave permission to use the tissue samples without individual consent in this retrospective study (Valvira Dnro 10041/06.01.03.01/2012).

### Tissue samples and immunohistochemistry

We collected formalin-fixed and paraffin-embedded surgical tissue samples from the archives of the Department of Pathology, and de-identified and analyzed the tissue samples anonymously. All histological slides were re-evaluated by an experienced pathologist who defined and marked areas representing the highest grade of the individual tumor. Four 1.0-mm cores from each tumor block, two from the invasive front, and two from the center of the tumor, were sampled and embedded in a new paraffin block by a semi-automatic tissue microarrayer (Tissue Arrayer 1, Beecher Instruments Inc., Silver Spring, MD, USA) as previously described [18]. Sections of 4 μm were cut and processed for immunohistochemistry.

Sections were fixed on slides and dried for 12 to 24 hours at 37°C, then they were deparaffinized in xylene and rehydrated through gradually decreasing concentrations of ethanol to distilled water. For antigen retrieval, sections were treated in a PreTreatment module (Lab Vision Corp., Fremont, CA, USA) in Tris-HCl (pH 8.5) and in Tris-EDTA (pH 9) buffer for 20 minutes at 98°C. Sections were stained in an Autostainer 480 (Lab Vision). Tissues were incubated with anti-human Prox1 Antibody (R&D Systems, Inc., Minneapolis, MN, USA; diluted to 1:1800 = 18 μg/ml) overnight at room temperature. For detection ImmPRESS HRP Polymer Detection Kit, Peroxidase, anti-goat IgG (Vector Laboratories, Burlingame, CA, USA) was used. Colon cancer tissue served as a positive control for staining.

### Scoring of immunoreactivity

PROX1 immunoreactivity was successfully scored in 273 tumors. We scored cytoplasmic PROX1 intensity in cancer cells as 0 to 3. Negative immunoreactivity was scored as 0, weak positivity 1, moderate positivity 2, and strong positivity 3. All samples were scored independently by two researchers (A.L. and J.H.) without knowledge of clinical status and outcome data. Samples with discordant scores were re-evaluated until consensus was reached. The highest score of the four cores served for further analysis.

### Statistical analyses

Associations between PROX1 positivity and clinicopathological variables were assessed by the chi-square test or Fisher’s exact test. Disease-specific survival was calculated from date of surgery to death from gastric cancer.

Survival curves were constructed according to the Kaplan-Meier method and compared with the log-rank test. For univariable and multivariable survival analysis, the Cox proportional hazard model had the following covariates entered: age, gender, stage, TNM classification, Lauréns classification, tumor size, and PROX1 expression. Stage, TNM classification, and tumor size were categorical covariates. A p-value of <0.05 was consider statistically significant. All statistical analyses were done with IBM SPSS Statistics version 22.0 (IBM Corporation, Armonk, NY, USA).

## Results

### Immunohistochemistry and association of PROX1 with clinicopathological variables

**Fig 1:**
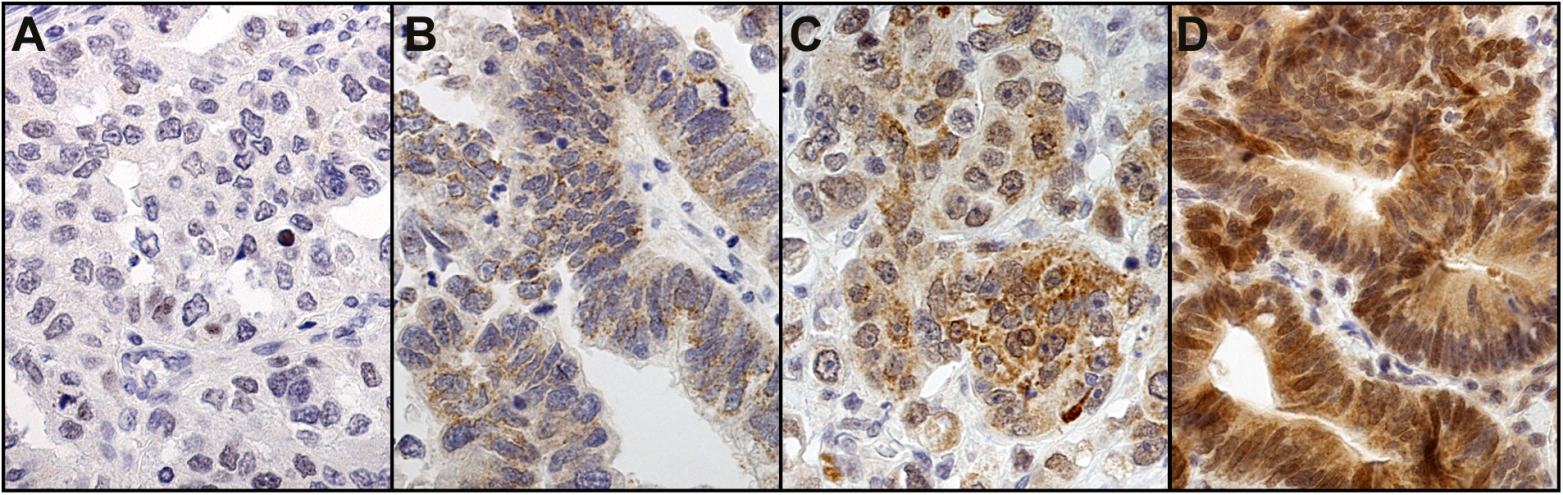
Representative images of PROX1 staining representing gastric cancer tumors with negative (A), weak (B), moderate (C), and strong (D) staining. Original magnification 40x.

Of 273 cases, the cytoplasmic PROX1 reactivity was weakly positive in 99 (36.3%), moderately positive in 39 (14.3%), strongly positive in 17 (6.2%), and negative in 118 (43.2%). In the final analysis, negative and weakly positive was regarded as low expression, and moderately and strongly positive as high expression. Representative images of immunostainings are presented in Fig 1. PROX1 staining occurred mainly in the cytoplasm, and in some strongly stained samples we noted also nuclear immunopositivity. Low PROX1 immunostaining associated with diffuse cancer type (p = 0.002; Table 1). No other clinicopathological variables associated with PROX1.

**Table 1:** Association of PROX1 with clinicopathological variables in gastric cancer patients.

	PROX1					
		Low		High		
Clinicopathological variable	n	n	%	n	%	p-value
**Age, years**						
<66	129	104	80.6	25	19.4	0.661
≥66	144	113	78.5	31	21.5	
**Gender**						
Male	130	100	76.9	30	23.1	0.317
Female	143	117	81.8	26	18.2	
**TNM stage**						
IA-IB	52	40	76.9	12	23.1	0.375
IIA-IIB	64	47	73.4	17	26.6	
IIIA-IIIC	100	81	81.0	19	19.0	
IV	56	48	85.7	8	14.3	
**pT classification**						
pT1	40	30	75.0	10	25.0	0.706
pT2	40	30	75.0	10	25.0	
pT3	85	69	81.2	16	18.8	
pT4	108	88	81.5	20	18.5	
**pN classification**						
pN0	90	66	73.3	24	26.7	0.246
pN1	37	28	75.7	9	24.3	
pN2	62	52	83.9	10	16.1	
pN3	76	64	84.2	12	15.8	
**pM classification**						
pM0	217	169	77.9	48	22.1	0.196
pM1	56	48	85.7	8	14.3	
**Laurén classification**						
Intestinal	111	77	69.4	34	30.6	0.002
Diffuse	154	134	87.0	20	13.0	
**Tumor size, cm**						
<5 cm	99	73	73.7	26	26.3	0.109
≥5 cm	167	137	82.0	30	18.0	

### Survival analyses

**Fig 2:**
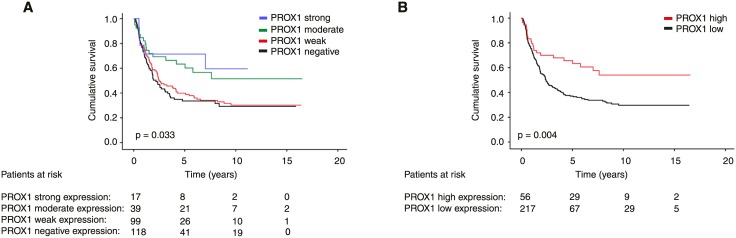
Gastric cancer-specific survival according to the Kaplan-Meier method, (A) cytoplasmic PROX1 immunoexpression and (B) grouped into high (strong and moderate staining) and low (weak and negative staining) expression. P-value for log-rank test.

In gastric cancer-specific survival analysis according to the Kaplan-Meier method patients with strong and the moderate immunostaining have clearly better survival than patients with weak or negative immunoexpression (p = 0.033, log-rank test; Fig 2A). In the final analysis, we combined strong and moderate immunoexpression to represent high expression and weak and negative immunoexpression to represent low expression. Gastric cancer patients with high PROX1 expression had a cancer-specific 5-year survival of 65.6% (95% CI 52.7–78.5), compared to 37.1% (95% CI 30.2–44.0) for patients with low expression (p = 0.004, log-rank test; HR = 0.52 (95% CI 0.33–0.82), Fig 2B, Table 2).

**Table 2:** Kaplan-Meier analysis for disease-specific survival stratified for subgroups of gastric cancer patients.

	5-year cumulative survival (95% CI)			
Subgroup	All patients	PROX1 low	PROX1 high	p-value
**PROX1**	43.3 (37.4–49.2)	37.1 (30.2–44.0)	65.6 (52.7–78.5)	0.004
**Age, years**				
<66	47.8 (39.6–56.0)	42.3 (32.5–52.1)	79.3 (63.0–95.6)	0.007
≥66	39.1 (31.1–47.1)	32.0 (22.6–41.4)	53.3 (34.5–72.1)	0.140
**Gender**				
Male	46.3 (37.7–54.9)	35.5 (25.1–45.9)	68.5 (51.4–85.6)	0.019
Female	40.9 (33.1–48.7)	38.1 (29.1–47.1)	62.1 (42.5–81.7)	0.095
**Laurén classification**				
Intestinal	52.0 (42.6–61.4)	40.0 (28.2–51.8)	73.7 (57.8–89.6)	0.025
Diffuse	36.7 (29.4–44.0)	35.7 (27.3–44.1)	50.0 (28.0–72.0)	0.290
**TNM stage**				
IA-IB	93.0 (86.3–99.7)	91.9 (83.1–100)	100	0.264
IIA-IIB	64.5 (52.5–76.5)	56.0 (40.7–71.3)	93.8 (81.8–100)	0.041
IIIA-IIIC	23.6 (15.4–31.8)	19.2 (10.2–28.2)	36.5 (13.2–59.8)	0.144
IV	5.7 (0–12.0)	2.4 (0–6.9)	25.0 (0–55.0)	0.745
**pT classification**				
pT1	93.6 (86.5–100)	93.2 (84.2–100)	100	0.275
pT2	77.1 (63.8–90.4)	81.0 (65.9–96.1)	75.0 (44.0–100)	0.789
pT3	32.5 (22.7–42.3)	26.0 (15.4–36.6)	71.4 (47.7–95.1)	0.029
pT4	18.1 (10.7–25.5)	9.9 (3.0–16.7)	40.0 (18.4–61.6)	0.067
**pN classification**				
pN0	73.8 (65.0–82.6)	67.2 (55.4–79.0)	91.5 (80.1–100)	0.169
pN1	45.0 (28.7–61.3)	35.0 (15.8–54.2)	77.8 (50.6–100)	0.081
pN2	36.3 (24.3–48.3)	32.5 (18.8–46.2)	66.7 (35.9–97.5)	0.092
pN3	15.8 (7.6–24.0)	13.9 (5.1–22.7)	16.7 (0–37.9)	0.957
**Tumor size, diameter**				
<5 cm	70.6 (62.0–79.2)	67.1 (56.1–78.1)	87.1 (73.4–100)	0.030
≥5 cm	25.6 (18.9–32.3)	20.0 (12.7–27.3)	47.9 (29.5–66.3)	0.111

CI = confidence interval

**Fig 3:**
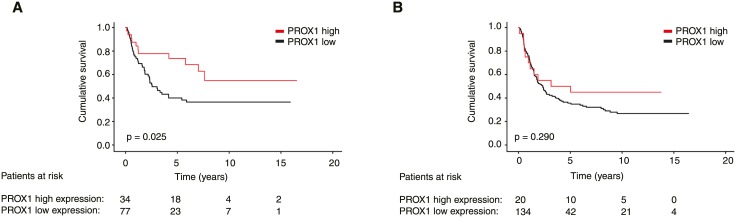
Expression of PROX1 in the subgroups of (A) intestinal and (B) diffuse cancer type in gastric cancer. P-value for log-rank test.

In subgroup analysis, PROX1 was a significant marker of better prognosis in patients aged under 66 (p = 0.007), among men (p = 0.019), and among patients with tumors of less than 5 cm in diameter (p = 0.030). PROX1 was a significant marker of better prognosis in the subgroup of intestinal cancer type (p = 0.025; Fig 3A) whereas it did not show a prognostic significance among patients with diffuse cancer type (p = 0.290; Fig 3B). In other subgroups studied PROX1 did not serve as a prognostic marker.

PROX1 expression remained significant in multivariable analysis (HR = 0.56; 95% CI 0.35–0.90; p = 0.017; Table 3). Other independent prognostic factors in multivariable analysis were advanced age and distant metastasis, and tumor size greater than 5 cm.

**Table 3:** Cox regression analysis for disease-specific survival of gastric cancer patients.

	Univariable survival analysis			Multivariable survival analysis		
Variable	Hazard ratio	95% CI	p-value	Hazard ratio	95% CI	p-value
**Age, years**						
<66	1.00			1.00		
≥66	1.38	1.02–1.84	0.034	2.45	1.69–3.57	<0.001
**Gender**						
Male	1.00			1.00		
Female	1.12	0.83–1.50	0.450	1.16	0.81–1.66	0.406
**TNM stage**						
IA-IB	1.00			1.00		
IIA-IIB	5.45	2.26–13.2	<0.001	3.22	0.82–12.6	0.093
IIIA-IIIC	15.7	6.83–35.9	<0.001	8.94	1.84–43.5	0.007
IV	46.1	19.6–109	<0.001	26.9	5.25–138	<0.001
**pT classification**						
pT1	1.00			1.00		
pT2	3.38	1.20–9.47	0.021	1.30	0.33–5.09	0.712
pT3	11.9	4.80–29.7	<0.001	1.89	0.44–8.06	0.392
pT4	19.0	7.71–47.0	<0.001	1.90	0.43–8.41	0.401
**pN classification**						
pN0	1.00			1.00		
pN1	2.43	1.41–4.20	0.001	0.87	0.44–1.74	0.701
pN2	3.20	2.02–5.09	<0.001	0.69	0.34–1.37	0.283
pN3	6.66	4.32–10.3	<0.001	1.19	0.60–2.35	0.615
**pM classification**						
pM0	1.00			1.00		
pM1	5.75	4.11–8.03	<0.001	26.9	5.25–138	<0.001
**Laurén classification**						
Intestinal	1.00			1.00		
Diffuse	1.24	0.96–1.61	0.101	1.31	0.94–1.84	0.113
**Tumor size**						
≤5 cm	1.00			1.00		
>5 cm	3.71	2.59–5.33	<0.001	1.64	1.05–2.56	0.030
**PROX1**						
Low	1.00			1.00		
High	0.52	0.33–0.82	0.005	0.56	0.35–0.90	0.017

CI = confidence interval

## Discussion

Here we show that cancer-specific 5-year survival was significantly better for gastric cancer patients with PROX1-positive tumors. In multivariable analysis, high PROX1 immunopositivity served as an independent marker of better prognosis. Among patients with intestinal cancer, those with high PROX1 immunostaining had superior survival.

PROX1 was expressed evenly throughout the cytoplasm, and in some strongly stained samples we noted also nuclear positivity. Earlier studies have reported both cytoplasmic and nuclear staining patterns [16,19]. The cytoplasmic PROX1 expression by immunohistochemistry correlates with *PROX1* mRNA amplification [19]. High mRNA levels correlate also with regional lymph node metastasis but not with distant metastasis. Depending on the cancer, the staining pattern varies. In colonic [4] and hepatocellular carcinomas [6] and in gliomas [15], the PROX1 staining is mainly nuclear, whereas in pancreatic cancer, mainly cytoplasmic staining is observed [9]. The role of cytoplasmic PROX1 expression still remains unknown. One hypothesis is that PROX1 is enriched and activated in the cytoplasm of the cell before being translocated to the nucleus to become functionally active [4].

In gastric cancer, Park et al. [16] studied PROX1 by silencing its expression in cell lines by small interfering RNA against *PROX1*. Cell proliferation was inhibited by *PROX1* knockdown, suggesting that PROX1 may play a role in regulating cell fate by reducing apoptosis as well as by promoting proliferation of gastric cancer cells. They also investigated the prognostic role of PROX1 in 327 gastric cancer patients by immunohistochemistry, finding that the survival of patients with PROX1-positive tumors was significantly worse than for those with PROX1-negative tumors. Unexpectedly, our result contradicted this. Reasons for the difference between their results and the present ones may be several. That 2017 Korean study did not include patients with metastasis. Our chosen antibody was anti-human Prox1 Antibody (R&D Systems Inc.), whereas they used a polyclonal rabbit anti-human PROX1 (Santa Cruz Biotechnology). They apparently stained whole tissue sections, whereas we utilized the TMA technique. As we scored cytoplasmic intensity in hot-spot tumor areas, they multiplied the stained area by the intensity score, and attributed a score more than or equal to 6 out of 9 for positive grouping. The effects of PROX1 expression on patient prognosis need to be further studied to determine its value as a prognostic tumor marker.

MicroRNAs (miRNAs) are involved in a variety of biological processes, and their dysregulation is linked to tumorigenesis and tumor progression. Earlier studies suggest an important role in tumor biology for miR-489. Zhang et al. [17] studied the expression levels of miR-489 in gastric cancer tissues and cell lines to investigate its role in the regulation of aggressive behavior of gastric cancer cells. They demonstrated that miR-489 is downregulated in human gastric cancer tissues and cell lines. Interestingly, miR-489 transfection reduces PROX1 protein in gastric cancer cells, and PROX1 silencing seems to inhibit proliferation. Further, PROX1 overexpression reverses miR-489-mediated suppression of proliferation and invasion of gastric cancer cells. In gastric cancer tissues, this study found a negative correlation between miR-489 and PROX1 protein expression. Zhang et al. suggest that PROX1 is a direct target of miR-489 in gastric cancer cells. As PROX1 depletion suppresses proliferation, one would expect that low PROX1 expression would correlate with better survival. In contrast, we noted the opposite result, because gastric cancer patients with high PROX1 expression had a better survival than did those with low or no expression.

Petrova et al. [5] in 2008 demonstrated similar findings in colon cancer. According to this study, PROX1 facilitates tumor progression by affecting cell adhesion and polarity. As a downstream effector of TCF/β-catenin signaling in colorectal cancer, loss of PROX1 does not directly affect cell proliferation, but it rather shifts the transcriptional phenotype of colon cancer cells towards more slowly growing adherent cells. Further, overexpression of PROX1 potentiates intestinal adenoma development, suggesting its important role in formation and growth of in situ lesions, which may progress to carcinoma. In addition to this oncogenic role of PROX1, it may in gastric cancer play several roles, as we found that high PROX1 expression was associated with a better prognosis.

The strength of this study is its large gastric cancer patient cohort with a long and reliable clinical follow-up and survival data. By the TMA method, only small areas of each tumor are evaluated, not whole-tissue sections. On the other hand, the TMA technique allows analysis of large patient cohorts with a homogenous staining method.

In conclusion, our study shows that PROX1 expression serves as a marker of favorable prognosis in gastric cancer.
